# Quality of intrapartum and newborn care in Tigray, Northern Ethiopia

**DOI:** 10.1186/s12884-019-2184-z

**Published:** 2019-01-18

**Authors:** Girmatsion Fisseha, Yemane Berhane, Alemayehu Worku

**Affiliations:** 1 Mekelle University, School of Public Health, P.O.BOX: 1871 Mekelle, Ethiopia; 2 Addis Continental Institute of Public Health, Addis Ababa, Ethiopia; 3 Addis Ababa University, School of Public Health, Addis Ababa, Ethiopia

**Keywords:** Quality, Intrapartum and newborn care, Factors associated, Northern Ethiopia

## Abstract

**Background:**

Quality intrapartum and newborn care is considered to be poor in Sub-Saharan Africa. However, studies done in Ethiopia are limited. Therefore, this study was conducted to assess the magnitude and factors associated with quality intrapartum and newborn care in Tigray, Northern Ethiopia.

**Methods:**

Facility based survey was conducted from December 2014 to February 2015 in Tigray, Northern Ethiopia. The quality of intrapartum care provided by a total of 106 skilled birth attendants to 216 labouring mothers and newborns were observed during childbirth in the health facilities. Standardized questionnaires and checklist were utilized to collect data. Quality of intrapartum service was measured using standard intrapartum criteria. Thus, good quality service was considered if the mother and newborn scored 75% or more of the intrapartum criteria during childbirth. Binary and multiple logistic regression model was used to determine the factors associated with quality intrapartum and newborn care services.

**Results:**

29.2% of mothers and 67.6% newborns received good quality care during intrapartum and immediate postpartum periods respectively. However, only 47.2% of mothers and newborns received a friendly care during childbirths. The independent predictors of quality intrapartum and newborn care were the appropriate use of partograph (AOR 3.92; 95% CI 1.78, 8.63), friendly maternal and newborn care services (AOR 7.9; 95% CI 3.59, 17.33), more than two years working experience (AOR 0.31; 95% CI 0.13, 0.73) and using services in different Zones in the study area.

**Conclusions:**

The quality intrapartum care is poor in the study area and it is associated with inappropriate use of partograph, unfriendly care, and experience of health providers. Scaling up obstetric service, continuous training and motivation of service providers and revising the criterion for accreditation of service providers are important.

**Electronic supplementary material:**

The online version of this article (10.1186/s12884-019-2184-z) contains supplementary material, which is available to authorized users.

## Background

Childbirth complications are the most common causes of death among women living in developing countries. The fetus and newborn are also at high risk of death, largely as the result of intrapartum hypoxia (i.e. “Birth asphyxia”) [1]. During this period, skilled birth attendants (SBAs) are considered as an effective intervention in improving quality service [2, 3]. Consequently, the government of Ethiopia has continued to scale up the number of SBAs and planned to reach to 80–90% SBAs utilization by 2020 [4]. The assumption is that quality could be improved by increasing the number of SBAs and health facilities. However, little emphasis has been given to the quality of intrapartum care provided by birth attendants.

Failure to provide quality maternal health service may lead to maternal deaths [5], with 99% of global maternal death occuring in developing countries [6]. Sub-Saharan Africa countries had the highest maternal mortality rate with poor progress in its reduction [6]. Ethiopia is one of the 10 countries with the highest maternal (412/100,000) [6, 7] and neonatal mortality statistics (37/1000 live births) since 2005 [8, 9].

As consequence of poor quality towards basic obstetric care, 49% of the total causes of perinatal deaths in Ethiopia were due to obstructed labor [10, 11]. Medical errors and poor hospital services are also the most common causes of maternal death in Tigray, where this study was conducted [12]. Therefore, a good proportion of pregnant women do not use maternal and childbirth health care services. Failure to utilize the above services by the women could be attributed either their negative attitude on quality or how care is provided [1]. Surprisingly, the health care system in Ethiopia is still in the same status of quality based on standards adopted from the 2003 WHO guidelines [1].

Limited studies have been conducted in Ethiopia on the associated factors and the level of quality intrapartum and newborn care. Very few studies have tried to assess the structural and outcome components of quality of care in African countries [13–16]. Although this study is from the same cohort of the recently published article [17] but the previous article was mainly focused on the overall quality of service delivery at health facility level. This focused on input, process and output quality measurements. However, the published article did not report on how the quality intraprtum and friendly care is given to mothers and newborns by health care providers [17]. Therefore, this study aimed to fill the above gap by assessing the magnitude and factors associated with quality intrapartum and newborn care. In addition the study attempted to explore the perspective of health facilities, service providers and mothers in relation to the quality of the services provided.

## Methods

The study was conducted in three zones of Tigray region, Northern Ethiopia. A facility based cross-sectional study design was used among skilled birth attendants (SBA) and mothers. Mothers with normal labour at the active first stage were observed until the immediate postpartum period. Mothers with complications during labour were excluded from this study.

A total of 106 skilled birth attendants and 216 labouring mothers from 32 health facilities were included in the study. Health facilities providing maternity services from each district were randomly selected. Sample size calculation for labouring mothers was determined by a single proportion of finite population with 95% confidence interval, marginal error (d) 5% and by taking 15% prevalence (P) of non-beneficial practice during childbirth in Ethiopia [18]. Adding 10% for non-response rate; a total of 216 mothers attending obstetric care were selected for the observation and chart review.

### Data collection procedures

Four types of data collection tools (non participatory observation, structured interview guide, facility audit and record review form) were used to gather data for this study. Non-participatory observation was used to observe mothers and SBAs during childbirth and immediate postpartum periods to assess quality intrapartum care. Mothers in active labour were observed during day and night time. The data collector stayed in the delivery room without interfering with the care being provided to mothers and newborns. A structured interview guide was used to conduct interviews with SBAs about their experiences and knowledge. Facility audit form was used to interview the head of the facilities about availability of essential equipment, drugs and supplies. A record review form was used to gather data from the mother charts about the completeness of the partograph. In addition to the training given, health professional who has more than two years working experience in the maternity ward were recruited to collect the data.

### Measurements

**Quality Intrapartum care:**The individual mothers’ score was categorized as receiving good quality if they scored 75% or more of the intrapartum criteria (69 or more of the 92 criteria). Detail of the measurement and tool is found in the recent published article [17]. All items were prepared as YES/NO questions (Attachment 1).

**Care during admission, first, second and third stage of labour, and immediate postpartum period:** Measured using items adapted from the WHO standards similar to the national guidelines [1, 19]. This is part of the intrapartum quality care. Mothers were categorized as receiving the standard quality care if they had received 17 or more of the 22 criteria during admission, 23 or more of the 31 criteria during the first stage of labour, 5 or more of the 6 criteria during the second stage of labour, 8 or more of the 10 criteria during the third stage of labour and 17 or more of the 23 criteria during immediate postpartum periods, corresponding to the 75th percentile of the process quality score distribution for all delivered mothers (Additional file 1).

**Quality newborn care:** was measured using 11 items adopted from the standards similar to the national guidelines [1, 19]. This is part of the intrapartum quality care. It was coded as 1 if mothers’ scores above 7 of the 11 criteria corresponding to 75th percentile and considered as receiving good quality care, otherwise, it was coded 0 (Additional file 1).

**Friendly mother and newborn care** was measured using 9 items adopted from FIGO [20]. It was coded as 1 if mothers’ scores above 6 of the 9 criteria corresponding to 75th percentile and considered as received good friendly care, otherwise, it was coded 0. This is part of the intrapartum quality care (Additional file 1).

**Knowledge of a skilled birth attendant** was measured using 17 items adapted from Reproductive Health Response in Conflict (RHRC) Consortium [21] which focused on basic and emergency obstetric care. If SBAs have scored above mean from the total 17 scores, he/she will be considered as having good knowledge, otherwise considered as poor (Additional file 2).

**Input quality** was measured using eight dimensions with a total of 40 items about facility preparedness. Facilities were categorized to be of good quality if it scored 30 or more of the 40 items. Detail of the measurement and tool is found in the recent publication [17].

**Misuse of partograph** was measured based on the time when skilled birth attendant start to fill partograph during labour and childbirth. Code 1 if skilled birth attendant starts and filled the partograph after birth, otherwise, coded as 0.

**Completeness of partograph:** measured using 14 items adopted from a component of a partograph. Completeness was assessed if > = 80% of the 14 items were filled correctly in the partograph to be classified as a complete partograph (> = 11 items out of 14 items), otherwise incomplete (Additional file 2).

### Statistical analysis

Data were analyzed using STATA version 12. Data were summarized by descriptive statistics like frequency distribution, percentage, tables, and graphs. Overall quality intrapartum, newborn care and friendly care were measured by aggregating data using cutoff point of 75%. In measuring quality care, the assumption was taken from a Malawi study [22]. The logistic regression model was used to assess the determinants of quality intrapartum and newborn service. A *p*-value of less than 0.05 was considered as the cutoff point for statistical significance. Hosmer–Lemeshow test was used to compare and rule out the goodness of fit of the models. Multicollinearity was examined, and all covariates having a value of variance inflation factor of 10 were tolerated.

## Results

A total of 106 SBAs and 216 labouring mothers visiting the 32 study health facilities during the study period were agreed to participate in the study. The mean age of the mothers involved in this study was 26.8 (SD ±6.2) years. Majority of the mothers, 192 (89%) were married and 77 (35.6%) were unable to read and write. Among the mothers included in this study, 69 (31.9%) had given birth for the first time and 200 (92.6%) had ANC follow-up. Most mothers, 148 (68.5%) used ambulance to reach the health facilities. Most mothers, 153 (70.8%) gave birth at the health center and 63 (29.2%) gave birth in hospitals. Of the 106 skilled birth attendants included in this study, 69 (65.1%) were diploma holder midwives and 80 (75.5%) were females. The median age and total working experience of attendants were 27 years (IQR: 24–35) and three years (IQR: 1–3), respectively (Table 1)**.**

**Table 1:** Socio-demographic characteristics of mothers and skilled birth attendants in Northern Ethiopia, 2015

Socio-demographic characteristics	Number	Percentage
A: Mothers characteristics (*n* = 216)		
Age (*n* = 216)		
< 18 years	4	1.8
18–23 years	71	32.9
24–29 years	61	28.2
30–35 years	60	27.8
> =36 years	20	9.3
Marital status		
Single	16	7.4
Married	192	89.0
Divorced	4	1.8
Separated	4	1.8
Educational status		
Unable to read and write	77	35.6
Able to read and write	9	4.2
1–4 grade	32	14.8
5–8 grade	39	18.1
9–10 grade	49	22.7
11–12 grade	10	4.6
Type of health facility used for delivery service		
Health center	153	70.8%
Hospital	63	29.2%
B: Skilled birth attendants characteristics (*n* = 106)		
Profession		
Midwife bachelor degree	34	32.1
Midwife diploma	69	65.1
Nurse diploma	3	2.8
Sex		
Male	26	24.5
Female	80	75.5
Age		
Less than 25 years	31	29.3
c25–30 years	40	37.7
c31–36 years	12	11.3
Greater than 36 years	23	21.7
Working experience		
< =1 year	46	43.4
2–4 years	42	39.6
> =5 years	18	17.0

According to the perspective of mothers, the reasons for coming to health facility was reported to being in close proximity to the health facility 24 (11.1%), difficult labour 54 (25%), bad birth outcome in pervious birth 4 (1.9%), advised by health providers to deliver at health facility 46 (21.3%) and to get quality delivery service 88 (40.7%).

### Quality of intrapartum care

**Fig. 1:**
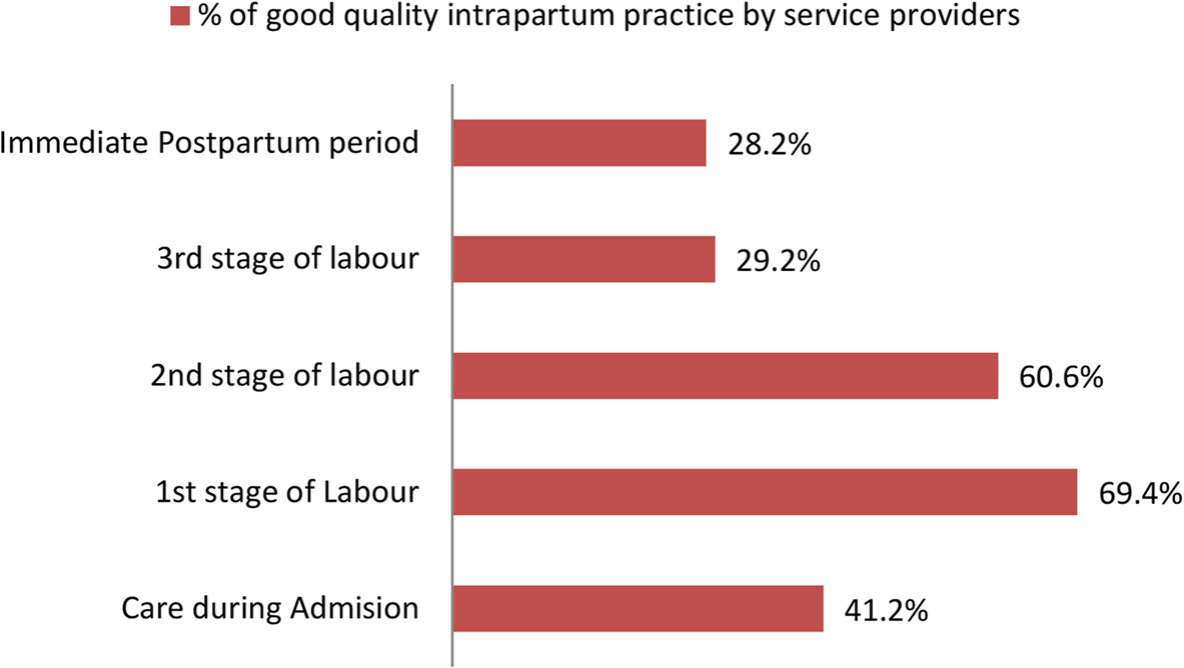
Intrapartum quality care from admission to immediate postpartum periods to mothers and newborn, Ethiopia, 2015

About 63 (29.2%, 95 CI: 23.1–35.2) of mothers received good quality intrapartum care. More than half, 146 (67.6%, 95 CI: 61.1–74.1) of newborns received good quality newborn care during delivery and the immediate postpartum period. One third of mothers got a quality intrapartum care during first-stage of labour 63 (29.2%) **(**Fig. 1).

**Fig. 2:**
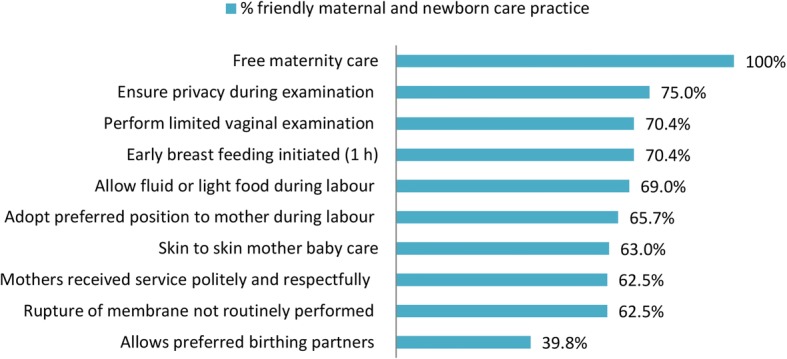
Friendly mother and newborn care during intrapartum and immediate postpartum period in Northern Ethiopia, 2015

Below half, 102 (47.2%, CI: 40.3–53.7) of mothers and newborns received friendly care during delivery and the immediate postpartum period. All mothers received maternity service free of charge and 40% of mothers were allowed to have birth companion (Fig. 2)**.**

### Factors associated with the good quality intrapartum service

After including variables at bivariate regression with *p*-value less than 0.25 into the multivariable logistic regression model; friendly care, appropriate use of partograph, working experience of health personnel and mother using service in different Zones in the study area were significant predictors of a good quality intrapartum and newborn care (Table 2).

**Table 2:** Factors associated with good quality intrapartum and newborn care in Northern Ethiopia, 2015

Variables	Quality intrapartum		Odd ratio with 95% CI	
	Good *n*(%)	Poor *n*(%)	Cured Odd ratio	Adjusted odd ratio
Friendly care during birth				
Poor	14(12.3%)	100(87.7%)	1	1
Good	49(48.0%)	53(52.0%)	6.6(3.34–13.05)**	7.9 (3.59–17.33)**
Misuse of partograph by health providers				
Yes	17(16.0%)	89(84.0%)	1	1
No	46(41.8%)	64(58.2%)	3.76(1.98–7.15)**	3.92(1.78–8.63)**
Service providers working experience				
< 1 Year	25(36.8%)	43(63.2%)	1	1
> =2 Years	38(25.7%)	110(74.3%)	0.59(0.32–1.1)	0.31 (0.13–0.73)*
Zone				
East	22(20.4%)	86(79.6%)	1	1
South east	24(51.1%)	23(48.9%)	4.08(1.95–8.54)**	6.2(2.36–16.28)**
South	17(27.9%)	44(72.1%)	1.5(0.73–3.13)	1.7(0.64–4.50)
Type of health facility care given				
Health center	49(32.0%)	104(68.0%)	1	1
Hospital	14(22.2%)	49(77.8%)	0.61(0.31–1.2)	0.54(0.21–1.41)
Sex of Health provider who provide care				
Male	17(34.0%)	33(66.0%)	1	1
Female	46(27.7%)	120(72.3%)	0.74(0.38–1.46)	1.34(0.56–3.24)
Knowledge of service providers on obstetric and newborn care				
Good	26(26.3%)	73(73.7%)	1	1
Poor	37(31.6%)	80(68.4%)	1.3(0.72–2.35)	1.91(0.87–4.19)
Health facility input quality (readiness)				
Good	45(25.6%)	131(74.4%)	1	1
Poor	18(45.0%)	22(55.0%)	2.38(1.17–4.84)*	1.48 (0.55–3.94)
Mode of current birth				
SVD	56(27.9%)	145(72.1%)	1	1
Instrumental	7(46.7%)	8(53.3%)	2.27(0.78–6.54)	1.25(0.33–4.79)

* Significant variable (*p*-value < 0.05), ** significant variable (*p*-value < 0.001), SVD: spontaneous vaginal delivery

## Discussion

The aim of this study was to assess the magnitude and factors associated with quality intrapartum care during childbirth and the immediate postpartum period. Accordingly, only 29.2% of mothers received good quality intrapartum care. Working experience, friendly maternal and newborn care, appropriate use of partograph, and using service at different Zones of the study area were significant predictors of quality intrapartum care.

In this study, the practice of quality intrapartum care was poor, thus only 29.2% of mothers received standard services during childbirth. This study result is consistent with studies done in some Sub-Saharan Africa [15, 16, 23], where the intrapartum care was reported to be poor. This finding is lower than the study done in Tanzania (60%) [24], and higher compared to another study in Tanzania (14%) [25]. The difference in the findings could be due to difference in measuring the quality care and the study subjects included. However, this study took a composite of variables to measure quality care and involved observations. This finding indicated that there is delay in treatment (if provider do not follow and provide service, according to the standards) which is the most common cause of death to the mother and newborn. Since intrapartum stillbirth and puerperal sepsis are often related to the poor intrapartum care [26].

The quality of newborn care in this study was found to be poor. Only 67.6% of newborns received standard care, based on the standards. This implies that service providers are neglecting the service to newborns, missing the procedures, or have poor skills to care for newborn. However, our finding is higher compared to the findings of two studies in Ghana which was (33%) [27] and (42%) [15] and another study in Ethiopia (18%) [18]. The difference could be due to the use of different measurement for quality newborn care between studies. In the Ghanaian study, the focus was more on the components of availability of materials for newborn care and emergency newborn care [15].

In the current study, mothers who received friendly care during childbirth were more likely to receive quality intrapartum care; this indicates there are mothers who neither received quality intrapartum nor friendly care during childbirths. The reasons could be due to poor skill and competency of service providers. In addition, inadequate number of skilled providers, high workload, and poor job satisfaction could be other reasons for the poor service [12, 14, 16]. The result of this study is consistent with studies in developing countries in which friendly service to mother during childbirth is poor [28, 29]. This may influence future maternal health service utilizations (antenatal, delivery and postnatal), and reduction of maternal and newborn mortalities.

Misuse of partograph during childbirth was one of the problems identified in this study. Those service providers who used and filled partograph appropriately were more likely to practice quality intrapartum care than those who misused. This indicated poor attitude, knowledge and skills on how to fill partograph among service providers. Similar problem was reported in Ethiopia [18]. This finding is consistent with studies in Africa, where the misuse of the partograph is common [15]. Not using or misusing of partograph can delay treatment in case of prolonged labour which leads to obstructed labour, ruptured uterus, bleeding, fetal death and infection to mothers and newborns [18].

In the current study, there was a significant difference in the quality of intrapartum care given in the three Zones. This difference could be due to the improper allocation of resources like mal-distribution of human resources, opportunity in training or involvement of non-governmental organizations in the three Zones. Coordination and monitoring by the local regional health bureau could improve the equitable distribution of resource between the Zones.

In this study, having more than two years working experience at maternity service was less likely associated with good quality intrapartum care. This indicates, whether a service providers work for many years or not, they are not improving their practice, this could be due to the lack of opportunity for appropriate training. This is supported by another study in sub-Saharan Africa where obstetric experience showed a non-linear relationship with knowledge and skills [30]. Refresher trainings and reform in human resource management is critical in order to improve skills and competency of health professionals in health facilities. It is also important to allocate adequate budget for training and for continuous professional career development.

Quality intrapartum care was not significantly associated with quality care services from health center or hospital. This indicates that childbirth care was provided poorly in both hospitals and health centers. Compared to periphery areas (health centers), the service given at hospitals is expected to be different and better in terms of quality cares due to the availability of experienced and high level human power, resource and supplies. However, in the current study, there is no difference between the care given in hospitals and health centers. This could be due to high workload of service providers as a result of high case follow at hospitals. So, it is important to give attention to the care given in hospitals.

As a limitation of this study, there may be observer bias between data collectors and Hawthorne effect. But during the lengthy period of observation, it is difficult for the health care personnel maintain their artificial standards of behavior during the long period of observation, and therefore any behavior change will be likely to persist during childbirth [31]. To reduce this effect, we tried to exclude the first observation from each skilled attendant. In addition, we recruited experienced skilled birth attendants, given intensive training and standardization of instrument prior to data collection and continuous supervision during data collection were done. The small sample size could also have an effect on the association between dependent and independent variables. This was due to the small case flow of mother’s in the study area, especially in rural health centers. Therefore, those limitations can under or over estimate the associations. Hence, it is better to consider those limitations while interpreting these findings.

This study did not relate the outcome variable (quality intrapartum care) with the morbidity and mortality during childbirth since most of the study areas were in rural health facilities. Many mothers in those facilities were immediately referred to higher institutions, in case of even minor complications, so it was difficult to follow these mothers till the end and difficult to see the birth outcome. Besides this, in case of the complications, the health providers use different standards in managing the complications and did not follow the usual guideline or checklist for normal birth. Therefore, to reduce this mix up in population (mother with normal labour and with complications), we only followed mothers with normal labour. Even after recruiting, we excluded the mothers with complications whatever the causes because it was difficult to follow the mothers in the same directions and using the same checklist. But, it is possible to relate quality intrapartum care with morbidity or mortality in hospital based research since it is less likely to refer the mothers to another facility. So, we recommend considering this in future studies conducted in hospitals.

## Conclusions

Quality intrapartum and newborn care is below the standard. Working experience among service providers, friendly maternal and newborn care, and appropriate use of partograph were the predictors of quality intrapartum and newborn care. Using service in hospital or health center was not associated with quality intrapartum and newborn care. The care given was also different in the three zones of the study areas. This shows there is poor adherence to standards, imbalance in qualified service providers, equipments and supplies in the three Zones. Therefore, renewing licenses, providing refresher training and on-site mentoring to service providers, staff motivation mechanism for overloaded skilled providers are essential. Giving emphasis to the quality care of the mother and newborn during childbirths at each level of health facility is also very important.

## Additional files

Additional file 1: List of variables used in measuring stages of intrapartum quality service, quality of newborn care, and friendly mother and newborn care in Northern Ethiopia. (PDF 637 kb)

Additional file 2: List of variables used in measuring knowledge of skilled birth attendant and completeness of partograph in Northern Ethiopia. (PDF 559 kb)

## Abbreviations

FIGO: International Federation of Gynecology and Obstetrics
SBAs: Skilled birth attendants
SVD: spontaneous vaginal delivery
