# Supplementary material for: Quality of intrapartum and newborn care in Tigray, Northern Ethiopia
Source: BMC Pregnancy Childbirth. 2019 Jan 18;19:37. doi: 10.1186/s12884-019-2184-z (PMC6339373; doi:10.1186/s12884-019-2184-z)
Supplement: Supplementary file 1 — List of variables used in measuring stages of intrapartum quality service, quality of newborn care, and friendly mother and newborn care in Northern Ethiopia. (PDF 637 kb) [file 12884_2019_2184_MOESM1_ESM.pdf]

**Table 1: Lists of variables used in measuring stages in Intrapartum and immediate postpartum quality in Northern Ethiopia study**

| **Variables used to meaure stage of qaulity intrapartum care:** | **Observed** | |
|---|---|---|
| **A. During Admission:** | | |
| Provider greets the woman and her companion (if present) in a cordial manner | Yes | No |
| Provider responds to mother immediate needs (thirst, hunger, cold/hot, need to urinate, | Yes | No |
| Provider ask mothers name | Yes | No |
| Provider ask her age | Yes | No |
| Provider ask about number of previous pregnancies/births | Yes | No |
| Provider ask any complications during labour and postpartum period | Yes | No |
| Provider ask other general medical problems | Yes | No |
| Provider ask use of medications | Yes | No |
| Provider ask about her HIV status | Yes | No |
| Provider estimate gestational age using last menstrual period | Yes | No |
| Provider ask mothers how frequently labour are occurring | Yes | No |
| Provider ask if her membranes ruptured: when, what color and what smell it had | Yes | No |
| Provider ask mothers whether she feels the baby's movements | Yes | No |
| Provider ask mothers when the painful regular contractions began | Yes | No |
| Provider ask mothers whether she has experienced vaginal bleeding, fever, severe headaches, blurred vision, convulsion, or if any other problems the client is concerned | Yes | No |
| Provider performs HIV test if status unknown | Yes | No |
| Provider records the information on clinical history chart | Yes | No |
| Ensures privacy with screen or curtain to separate the women from others at least during examination | Yes | No |
| Washes hands with running water and soap for 10-15 seconds and dries with an individual clean towel or allows hands to air dry | Yes | No |
| Explain each step | Yes | No |
| At least one encourage the woman to ask questions | Yes | No |
| Respond to the woman/ companion questions politely and in respectful manner | Yes | No |
| **B. First stage of labour:** | | |
| Providers takes mother temperature | Yes | No |
| Provider takes mother pulse | Yes | No |
| Provider measures mother blood pressure | Yes | No |
| Provider determines mother respiratory rate | Yes | No |
| Provider measures mother fundal height | Yes | No |
| Provider determines foetal lie and presentation | Yes | No |
| Provider identifies degree of decent by abdominal palpation (from five to zero fingers above the pubis) | Yes | No |
| Provider evaluates uterine contractions (frequency and duration over a 10- minute period) | Yes | No |
| Provider Auscultates foetal heart rate (FHR) | Yes | No |
| **During physical examination, providers:** | | |
| Puts sterile gloves on both hands | Yes | No |
| Carefully inserts two fingers of the exam hand | Yes | No |
| Assess cervical examination | Yes | No |

| | | |
|---|---|---|
| Assesses molding | Yes | No |
| Assesses station of presenting part | Yes | No |
| Assesses position | Yes | No |
| Gloves are removed after being immersed in 0.5% chlorine solution and placed in a leak-proof container | Yes | No |
| Records all information on the clinical records | Yes | No |
| Provider start partograph to follow progress of labour | Yes | No |
| **Infection prevention practice of providers:** | | |
| Cleanse the vulva with antiseptic solution before performing vaginal examination | Yes | No |
| Performs limited vaginal examination (e.g, every four hours or indicated) | Yes | No |
| Uses sterile gloves when performing vaginal examination or when in contact with body fluids | Yes | No |
| Rupture of membranes is not performed routinely | Yes | No |
| **Provider prepares to assist the birth:** | | |
| Has the delivery pack and other essential material and ready to assist the delivery (delivery set, episiotomy set, newborn resuscitation set, glove, delivery gown, apron, mask) | Yes | No |
| Prior to delivery, washes hands with running water and soap for 10-15 seconds and dries an individual clean towel or allows hands to air dry | Yes | No |
| Puts sterile double gloves on hands | Yes | No |
| **Provider:** | | |
| At least once encourage woman to walk around | Yes | No |
| At least once encourage the woman to change position according to the desire and comfort | Yes | No |
| At least once encourage the woman to take light food or drink fluid in labour | Yes | No |
| Allow the woman to have her preference companion at labour room | Yes | No |
| Provide emotional support to woman during labour and delivery | Yes | No |
| At least once encourages her to empty her bladder | Yes | No |
| **C. Second stage of labour: provider** | | |
| Holds the baby by the trunk and places the baby on a clean dry towel on the mother's abdomen | Yes | No |
| Dries baby vigorously and changes wet towel for a clean dry one | Yes | No |
| Ties or clamps cord when pulsations stop, or by 2-3 minutes after birth (no immediately after birth) | Yes | No |
| Cuts/clamps the umbilical cord using sterile scissors under gauze to prevent blood spurting | Yes | No |
| If the baby is breathing normally, passes the baby to mother for skin-to-skin contact on breast | Yes | No |
| Note the time of birth and records on partograph or in other records | Yes | No |
| **D. Third stage of labour: provider,** | | |
| Palpate the mother's abdomen to rule-out the presence of a second baby | Yes | No |
| Tells the woman that she will receive an injection and administers 10 IU of oxytocin IM or 600 mcg misprostol orally within 1 minute of delivery | Yes | No |
| Place the other hand on the women's symphysis pubis (over the sterile towel) | Yes | No |
| Maintain firm traction on the cord and waits for the uterus to contract | Yes | No |
| Upon contraction, applies firm and sustained downward traction on the cord with counter traction above the pubis to guard the uterus, until the placenta is expelled | Yes | No |
| If this maneuver does not provide immediate results, stops applying traction, holding the cord and clamp until the next contraction | Yes | No |
| Repeats control cord traction during contraction while simultaneously applying counter | Yes | No |

| | | |
|---|---|---|
| traction above pubis to guard uterus | | |
| With both hands, assists in the expulsion of the placenta by turning it over in the hands without applying traction twisting the membranes | Yes | No |
| Massage the uterus with one hand one sterile cloth over the abdomen until it contracts firmly | Yes | No |
| Checks to see whether the placenta is complete (maternal and foetal sides, plus membranes, lobs) | Yes | No |
| **E. Immediate postpartum periods: provider,** | | |
| Make sure that the woman is comfortable (clean, hydrated and warmly covered) | Yes | No |
| Ensures that the baby is well covered | Yes | No |
| Administer vitamin K to newborn | Yes | No |
| Provides tetracycline eye ointment 1% prophylaxis to newborn | Yes | No |
| Observe breast feeding initiated within the first hour after birth | Yes | No |
| Weight the baby | Yes | No |
| Discards the placenta in a leak-proof container with a plastic liner | Yes | No |
| Disposes of medical waste (gauze, etc) in a plastic container with a plastic liner | Yes | No |
| Puts the soiled linen in a leak-proof container | Yes | No |
| Places all reusable instruments in a 0.5% chlorine solution for 10 minutes | Yes | No |
| Disposes needle and siring in a puncture-resistance container, without removing, recapping or breaking the needle | Yes | No |
| Gloves are removed after being immersed in 0.5% chlorine solution and placed in a leak-proof container | Yes | No |
| Washes hands with running water and soap for 10-15seconds and dries with an individual clean towel or allows hands to air dry | Yes | No |
| **Provider monitors the mother and newborn after birth:** | | |
| Baby's breathing condition and breastfeeding | Yes | No |
| Vaginal bleeding | Yes | No |
| Bladder distension | Yes | No |
| Blood pressure | Yes | No |
| Uterine contraction | Yes | No |
| Pulse | Yes | No |
| Consciousness | Yes | No |
| Assists the woman with breastfeeding | Yes | No |
| Asks the woman if she has urinated and encourages her to do so whenever she wishes | Yes | No |
| Records the information on the women's clinical record and reports any abnormalities | Yes | No |

**Table 2: Lists of variables used in measuring quality newborn care during immediate postpartum quality in Northern Ethiopia study**

| Qno | Observe whether skilled birth attendant do the following after birth for the newborns | Response (observed) | Remark |
|---|---|---|---|
| | Skilled personnel: | | |
| 1 | Dries newborn baby vigorously and change wet towel immediately after birth | 1. Yes 2. No | |
| 2 | Evaluate breathing of newborn | 1. Yes 2. No | |
| 3 | Tie or clamp cord after 2-3 minute of birth | 1. Yes 2. No | |
| 4 | Ensure the baby is covered | 1. Yes 2. No | |
| 5 | Ensure the baby is in skin to skin contact with mother | 1. Yes 2. No | |
| 6 | keep newborn in the same room with mother | 1. Yes 2. No | |
| 7 | Ensure baby is start breastfeeding within 1 hour | 1. Yes 2. No | |
| 8 | Administered TTC eye ointment | 1. Yes 2. No | |
| 9 | Administered vitamin K | 1. Yes 2. No | |
| 10 | Weight the newborn baby | 1. Yes 2. No | |
| 11 | Check newborn breathing and breast feeding status every 15 minutes | 1. Yes 2. No | |

**Table 3: Lists of variables used in measuring friendly mother and newborn care in Northern Ethiopia study**

| Qno | Observe whether skilled birth attendant do the following after birth to mother and newborns | Response (observed) | Remark |
|---|---|---|---|
| 1 | Ensure privacy during labour | 1. Yes 2. No | |
| 2 | No physical emotionally and verbally abused | | |
| 3 | Evidence based practice (eg. Limited vaginal examination and no rupture of member routinely) | 1. Yes 2. No | |
| 4 | Adopt preferred position to mother during labour | | |
| 5 | Allow fluid or light food during labour | 1. Yes 2. No | |
| 6 | Allow preferred birthing partner | 1. Yes 2. No | |
| 7 | Skin to skin mother baby care | 1. Yes 2. No | |
| 8 | Early breast feeding with in 1 hr | 1. Yes 2. No | |
| 9 | Affordable or free maternity care | 1. Yes 2. No | |
