# Supplementary material for: Quality of intrapartum and newborn care in Tigray, Northern Ethiopia
Source: BMC Pregnancy Childbirth. 2019 Jan 18;19:37. doi: 10.1186/s12884-019-2184-z (PMC6339373; doi:10.1186/s12884-019-2184-z)
Supplement: Supplementary file 2 — List of variables used in measuring knowledge of skilled birth attendant and completeness of partograph in Northern Ethiopia. (PDF 559 kb) [file 12884_2019_2184_MOESM2_ESM.pdf]

Table 1: List of variable used to measure skilled birth attendants knowledge on basic and emergency obstetric care

**Knowledge question on basic and emergency obstetric care**				
**Essential obstetric care**		**Response**	**Code**	**Answer**
1	How did you establish the patient was in labor?	1. Regular uterine contraction<br>2. Cervical dilatation<br>3. Show (bloody mucous discharge)<br>4. Breakage of water/ruptured membranes<br>5. Other (specify):	0<br>1	**Essential signs**<br>If variables **1**, **2** are both mentioned enter **1** in box below. Enter **0** if **less** than the essential two are entered
2	What observations or monitoring do you normally carry out during labour?	1. Monitor fetal heart rate pattern<br>2. Assess degree of molding<br>3. Assess cervical dilatation<br>4. Assess descent of head<br>5. Monitor uterine contractions<br>6. Monitor maternal blood pressure<br>7. Monitor maternal respiratory rate<br>8. Monitor maternal temperature<br>9. Monitor maternal pulse<br>10. Check the urine 11. Other (specify):	0<br>1	**Essential actions**<br>If variables**1**, **2**, **3**, **4**, **5** and **6** are all mentioned enter **1** in box below.<br>Enter **0** if **less** than the essential six are entered
3	Where do you normally record these observations?	1. On a partograph<br>2. In patients' notes<br>3. On partograph and patients' notes<br>4. On antenatal card<br>5. On a piece of paper 6. Other (specify):	0<br>1	**Essential action**<br>If variable **1** is mentioned enter **1** in box below.<br>Enter **0** if **variable one** not mentioned
4	Last time you attended to a delivery, what was the immediate care you gave to the newborn?	1. After birth of head, wipe face, nose, mouth<br>2. Cord care (sterile cut 4-6cm / umbilicus)<br>3. Ensure baby is breathing<br>4.Thermal protection<br>5. Breastfeeding initiated within one hour<br>6. Assess/examine newborn within one hour<br>7. Baby weighed<br>8. Eye prophylaxis	0<br>1	**Essential actions**<br>If variables**1**, **2**, **3** and **4** are all mentioned enter **1** in box below.<br>Enter **0** if **less** than the essential six are entered
5	When a woman comes with or develops heavy bleeding after delivery, what signs do you look for?	1. Uncontracted uterus<br>2. Signs of shock (dizziness, low BP)<br>3. Amount of external bleeding<br>4. Retained products/placenta<br>5. Check if bladder is full<br>6. Genital tract injuries<br>7. Signs of anemia<br>8. Other (specify):	0<br>1	**Essential actions**<br>If variables**1**, **2**, **3**, **4** and **5** are all mentioned enter **1** in box below.<br>Enter **0** if **less** than the essential six are entered
6	When a woman comes with or develops heavy bleeding after delivery, what action do you take?	1. Massage the fundus<br>2. Empty the woman's bladder<br>3. Give ergometrine im or iv (Oxytocin)<br>4. Start IV fluids<br>5.Take blood for Hb, and X-matching<br>6. Examine the woman for lacerations<br>7. Manual removal of retained products<br>8. Refer<br>9. Raise foot of bed<br>10. Other (specify):	0<br>1	**Essential actions**<br>If variables**1**, **2**, **3** and **4** are all mentioned enter **1** in box below.<br>Enter **0** if **less** than the essential six are entered
7	When a woman you have just delivered has a retained	1. Empty urinary bladder<br>2. Apply controlled cord traction	0<br>1	**Essential actions**<br>If variables**1**, **2**, **3** and **4** are

	placenta, what actions do you take?	3. Repeat oxytocics<br>4. Apply manual removal of the placenta<br>5. Give IV fluids<br>6. Monitor vital signs for shock & act<br>7. Check uterus is well contracted<br>8.Test blood for group and cross match<br>9. Prepare for theater<br>10. Refer 11. Other (specify):		all mentioned enter **1** in box below.<br>Enter **0** if **less** than the essential six are entered
8	When a woman comes with general malaise 48 hours after delivery, what signs do you look for?	1. High pulse<br>2. High fever<br>3. Septic shock (unrecordable BP)<br>4. Sub-involuted tender uterus<br>5. Foul smelling lochia<br>6.Tender abdomen	0<br>1	**Essential actions**<br>If variables**1**, **2**, **3**, **4** and **5** are all mentioned enter **1** in box below.<br>Enter **0** if **less** than the essential six are entered
9	When a woman complains of maliase 48 hours after delivery, what do you do?	1. Start IV fluids<br>2. Give parenteral antibiotics before referral to doctor or hospital<br>3. Administer analgesics/antipyretic<br>4.Take blood for BS & give malaria prophylaxis in endemic areas<br>5. Palpate abdomen<br>6. Examine lochia, perineum and breasts<br>7. Refer	0<br>1	**Essential actions**<br>If variables**1**, **2**, **3** and **4** are all mentioned enter **1** in box below.<br>Enter **0** if **less** than the essential six are entered
10	What danger signs would you look for if a pregnant woman comes with suspected severe malaria?	1. High temperature (above 38C)<br>2. Confusion/Coma<br>3. Pallor<br>3. Jaundice (yellow eyes)<br>4. Status of the fetus<br>5. Dizziness<br>6. Joint pains<br>7. Dehydration	0<br>1	**Essential actions**<br>If variables**1**, **2**, **3** and **4** are all mentioned enter **1** in box below.<br>Enter **0** if **less** than the essential six are entered
11	What are the signs for asphyxia neonatorum?	1. Heart rate less 100BPM<br>2. Gasping respiration<br>3. Slight muscle flexion<br>4. Poor or no reflexes<br>5. Blue to pale color	0<br>1	**Essential actions**<br>If variables**1**, **2**, **3, 4** and **5** are all mentioned enter **1** in box below.<br>Enter **0** if **less** than the essential six are entered
12	What are the signs and symptoms of infection in the newborn (sepsis)?	1. Poor or no breastfeeding<br>2. Hypothermia or hyperthermia<br>3. Restlessness or irritability<br>4. Respiratory difficulty<br>5. Foci of infection may be found in throat, skin, eyes<br>6. No apparent source of infection	0<br>1	**Essential actions**<br>If variables**1**, **2**, **3** and **4** are all mentioned enter **1** in box below.<br>Enter **0** if **less** than the essential six are entered
13	When a newborn is presented with signs of infection what action do you take?	1.Continue breastfeeding topped with<br>2.EBM via NG tube if necessary<br>3. Keep the baby warm<br>4. Keep airway clear<br>5. Start antibiotics and refer<br>6. Explain situation/condition to the mother or caregiver	0<br>1	**Essential actions**<br>If variables**1**, **2**, **3** and **4** are all mentioned enter **1** in box below.<br>Enter **0** if **less** than the essential six are entered
14	When a newborn is less than 2.5 kgs, what extra care do you provide?	1. Ensure thermal protection (skin-to-skin, etc.)<br>2. Provide extra support to mother to establish and maintain breastfeeding<br>3. Monitor baby closely for first 24 hours	0<br>1	**Essential actions**<br>If variables**1**, **2**, **3** and **4** are all mentioned enter **1** in box below.

		4. Ensure infection prevention<br>5. Monitor sucking capability<br>6. Refer to hospital		Enter **0** if **less** than the essential six are entered
15	What procedures are no longer carried out routinely during labor and delivery?	1. Artificial rupture of membranes<br>2. Episiotomies<br>3. Shaving<br>4. Routine suction of newborn babies<br>5. Bathing the baby within 24 hours<br>6. Milking of cord<br>7. Enema	0<br>1	**Essential Actions**<br>Enter 1 in box if 3 or more are mentioned.<br>Enter **0** if **less** than 3 are mentioned.
16	What actions should you take if a mother come with obstructed labour	1. Start IV fluid<br>2. Give parenteral antibiotics<br>3. Call doctor or refer<br>4. Monitor vital signs<br>5. Prepare for cesarean section<br>6. Take blood for Hb, group, and X match<br>7. Drain bladder by catheter<br>8. Record observations on partograph	0<br>1	**Essential Actions**<br>If variables **1, 2** and **3** are all mentioned enter 1 in box below.<br>Enter 0 if less than the essential three are entered
17	What actions should you take if a mother come with sever preeclampsia or eclampsia	1. Place in side-lying position<br>2. Protect from injury and spatula insidemoth<br>3. Give magnesium sulfate<br>4. Provide antihypertensives<br>5. Administer oxygen at 4–6 L per minute<br>6. Refer	0<br>1	**Essential Actions**<br>If variables **1, 2** and **3** are all mentioned enter 1 in box below.<br>Enter 0 if less than the essential three are entered

Table 2: List of questions used to measure completeness of partograph during the progress of labour.

s.no	The provider uses the partograph to monitor labour: observe, whether the provider completes partograph consistently	Resposne	Remark
A	***Records partograph information consistently***		
1	Records fetal heart rate every half hour	1. Yes 2. No	
2	Records maternal pulse rate every half hour	1. Yes 2. No	
3	Records strength and frequency of uterine contraction every 30min	1. Yes 2. No	
4	Records Blood pressure every four hours	1. Yes 2. No	
5	Records temperature every four hours	1. Yes 2. No	
6	Records vaginal examination every four hours	1. Yes 2. No	
7	Records fetal descent/station every four hours	1. Yes 2. No	
8	Records cervical dilatation and position every four hours	1. Yes 2. No	
9	At every vaginal examination checks amniotic fluids and status of membrane	1. Yes 2. No	
10	At every vaginal examination checks the degree of molding	1. Yes 2. No	
B	***Records partograph information completely***		
11	Records client name, gravid and parity	1. Yes 2. No	
12	Date and time of admission	1. Yes 2. No	
13	Records time of rupture of membrane	1. Yes 2. No	
14	Records all drugs, IV fluids she is taking	1. Yes 2. No	
